# Gossypol decreased cell viability and down-regulated the expression of a number of genes in human colon cancer cells

**DOI:** 10.1038/s41598-021-84970-8

**Published:** 2021-03-15

**Authors:** Heping Cao, Kandan Sethumadhavan, Fangping Cao, Thomas T. Y. Wang

**Affiliations:** 1 United States Department of Agriculture, Agricultural Research Service, Southern Regional Research Center, 1100 Robert E. Lee Boulevard, New Orleans, LA 70124 USA; 2 Beijing Forestry University, No. 35 Tsinghua East Road, Haidian District, Beijing, 100083 China; 3 United States Department of Agriculture, Agricultural Research Service, Beltsville Human Nutrition Research Center, 10300 Baltimore Ave, Beltsville, MD 20705 USA

**Keywords:** Biochemistry, Cancer, Plant sciences, Molecular medicine

## Abstract

Plant polyphenol gossypol has anticancer activities. This may increase cottonseed value by using gossypol as a health intervention agent. It is necessary to understand its molecular mechanisms before human consumption. The aim was to uncover the effects of gossypol on cell viability and gene expression in cancer cells. In this study, human colon cancer cells (COLO 225) were treated with gossypol. MTT assay showed significant inhibitory effect under high concentration and longtime treatment. We analyzed the expression of 55 genes at the mRNA level in the cells; many of them are regulated by gossypol or ZFP36/TTP in cancer cells. BCL2 mRNA was the most stable among the 55 mRNAs analyzed in human colon cancer cells. GAPDH and RPL32 mRNAs were not good qPCR references for the colon cancer cells. Gossypol decreased the mRNA levels of DGAT, GLUT, TTP, IL families and a number of previously reported genes. In particular, gossypol suppressed the expression of genes coding for CLAUDIN1, ELK1, FAS, GAPDH, IL2, IL8 and ZFAND5 mRNAs, but enhanced the expression of the gene coding for GLUT3 mRNA. The results showed that gossypol inhibited cell survival with decreased expression of a number of genes in the colon cancer cells.

## Introduction

**Figure 1:**
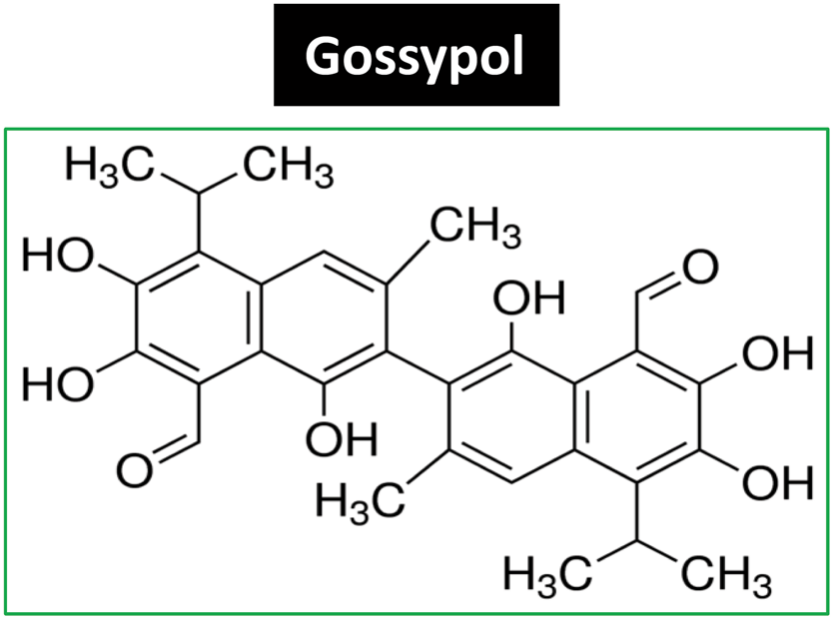
Chemical structure of gossypol (molar mass: 518.56 g/mol) (Image was from public domain Gossypol—Wikipedia).

Gossypol is a plant polyphenol with a highly colored yellow pigment (Fig. 1). It is found in the small intercellular pigment glands in the leaves, stems, roots, and seed of cotton plants (*Gossypium hirsutum* L.)^1^. Gossypol has traditionally been regarded as an anti-nutritional toxic compound. It has been known for a long time that consumption of gossypol-containing cottonseed oil contributes to its toxicity causing male infertility^2^. Therefore, gossypol is regarded as unsafe for most animal and human consumption. The residual gossypol in cottonseed meals limits its use primarily to feed ruminants, which have a relative high tolerance for the toxic compound^2–6^. Significant efforts have been conducted to reduce gossypol content in cottonseed by selecting glandless cotton varieties^7–12^ and genetic engineering of gossypol-free seeds of cotton plants^13–15^.

Gossypol was proposed recently to have potential biomedical applications. Gossypol and related compounds were reported to have anticancer activities associated with breast cancer^16–18^, colon cancer^19^, pancreatic cancer^20,21^ and prostate cancer^22,23^. It has antiobesity activities^16,24^, antiinflammatory activities^25,26^ and antifungal activities^27,28^. These new discoveries have generated intensive interest in biomedical field and enormous amounts of research have been directed at understanding the medical utilization of gossypol and related compounds.

Colon cancer is one of the deadliest diseases in the US and the World. American Cancer Society estimated that the lifetime risk of developing colorectal cancer is approximately 4.49% for men and 4.15% for women; which may cause 51,020 deaths during 2019 (https://www.cancer.org/cancer/colon-rectal-cancer/about/key-statistics.html). Some studies have explored the effect of gossypol and its analogs on colon cancer cells. They bind to and inactivate BH3 domain-containing antiapoptotic proteins^29^. Gossypol analog Ch282-5 (2-aminoethanesulfonic acid sodium-gossypolone) exhibits anti-proliferative and pro-cell death activity against colon cancer cells both in vitro and in vivo, and the response of colon cancer cells to the drug correlates with Ch282-5′s inhibition of anti-apoptotic Bcl-2 proteins, induction of mitochondria-dependent apoptotic pathway, and disruption of mitophagy and mTOR pathway^29^. It was also shown that (-)-gossypol is a potential small molecule inhibitor of MSI1-RNA interaction, and may be incorporated into an effective anti-cancer strategy^30^. A novel water-soluble gossypol derivative increases chemotherapeutic sensitivity and promotes growth inhibition in colon cancer^31^. Gossypol sensitizes the antitumor activity of 5-FU through down-regulation of thymidylate synthase in human colon carcinoma cells^32^. These studies have provided a promising beginning to use gossypol in the prevention and treatment of colon cancer. However, not enough was done on the regulation of gene expression at the mRNA level by gossypol in colon cancer cells, especially related to cytokine expression regulated by the anti-inflammatory zinc finger protein 36/tristetraprolin (ZFP36/TTP). Much more information is required to develop an effective strategy to combat colon cancer.

It is our aim to uncover more profound effects of gossypol on regulating the cell viability and expression of a wide range of target genes involved in cancer development. In this study, we analyzed the cell viability and expression of 55 genes (Table 1) whose expression is regulated by gossypol in cancer cells^20,33–39^ and macrophages^40^ or regulated by ZFP36/TTP in tumor cells^41–49^ and macrophages^50,51^. Human colon cancer cells (COLO 225) were treated with multiple concentrations of gossypol followed by quantitative PCR analysis. Our results showed that gossypol inhibited cell viability and suppressed a number of gene expression in the human colon cancer cells.

**Table 1:** Human qPCR primers.

ID	mRNA	Name	Forward primer (5′ to 3′)	Reverse primer (5′ to 3′)	Reference
H1	Ahrr1	Aryl hydrocarbon receptor repressor	AGGCTGCTGTTGGAGTCTCTTAA	CGATCGTTGCTGATGCATAAA	TTP^45^
H2	Bcl2	B-cell lymphoma 2	CAGCATGCGGCCTCTGTT	GGGCCAAACTGAGCAGAGTCT	Gossypol^37^
H3	Bcl2l1	B-cell lymphoma 2 like 1	GTGCGTGGAAAGCGTAGACA	ATTCAGGTAAGTGGCCATCCAA	TTP^94^
H4	Bnip3	BCL2 protein-interacting protein 3	GTCAAGTCGGCCGGAAAATA	TGCGCTTCGGGTGTTTAAAG	Gossypol^20^
H5	Cd36	Cluster of differentiation 36/fatty acid translocase	CTCTTTCCTGCAGCCCAATG	TTGTCAGCCTCTGTTCCAACTG	TTP^95^
H6	Claudin1	Maintain tissue integrity and water retention	GACAAAGTGAAGAAGGCCCGTAT	CAAGACCTGCCACGATGAAA	TTP^49^
H7	Cox1	Cyclooxygenase 1	CGCCCACGCCAGTGA	AGGCCGAAGCGGACACA	TTP^96^
H8	Cox2	Cyclooxygenase 2	CGATTGTACCCGGACAGGAT	TTGGAGTGGGTTTCAGAAATAATTT	TTP^48^
H9	Csnk2a1	Casein kinase 2 alpha 1	AGCGATGGGAACGCTTTG	AAGGCCTCAGGGCTGACAA	TTP^46^
H10	Ctsb	Cathepsin B	GACTTGTAGCTGCTGTCTCTCTTTGT	CAAGAGTCGCAAGAACATGCA	TTP^97^
H11	Cxcl1	Chemokine (C-X-C motif) ligand 1	GCCCAAACCGAAGTCATAGC	TGCAGGATTGAGGCAAGCT	TTP^98^
H12	Cyclind1	Cyclin D1	ACACGCGCAGACCTTCGT	CCATGGAGGGCGGATTG	Gossypol^38^
H13	Cyp19a1	Cytochrome P450 family 19 subfamily A member 1	GACATTGCAAGGACAGTGTGTTG	AGTCTCATCTGGGTGCAAGGA	Gossypol^35^
H14	Dgat1	Diacylglycerol O-acyltransferase 1	ACCTCATCTGGCTCATCTTCTTCTA	CCCGGTCTCCAAACTGCAT	DGAT^99,100^
H15	Dgat2a	Diacylglycerol O-acyltransferase 2a	CCCAGGCATACGGCCTTA	CAACACAGGCATTCGGAAGTT	DGAT^100,101^
H16	Dgat2b	Diacylglycerol O-acyltransferase 2b	ACTCTGGCCCTTCTCTGTTTTTTA	TCCACCTTGGTTGGGTGTGT	DGAT^100,101^
H17	E2f1	E2F transcription factor 1	CGGCGCATCTATGACATCAC	CAGCCACTGGATGTGGTTCTT	TTP^44^
H18	Elk1	ETS transcription factor	CTCCTCCGCATCCCTCTTTAA	AGCGTCACAGATGGGTCCAT	TTP^42^
H19	Fas	Fas cell surface death receptor	GAACTCCTTGGCGGAAGAGA	AGGACCCCGTGGAATGTCA	Gossypol^34^
H20	Gapdh	Glyceraldehyde-3-phosphate dehydrogenase	GGGTGTGAACCATGAGAAGTATGA	GGTGCAGGAGGCATTGCT	^83^
H21	Glut1	Glucose transporter 1	TGCTCATGGGCTTCTCGAA	AAGCGGCCCAGGATCAG	GLUT^51^
H22	Glut2	Glucose transporter 2	GCATTTTTCAGACGGCTGGTA	GCGCCAACTCCAATGGTT	GLUT^51^
H23	Glut3	Glucose transporter 3	GAGGATATCACACGGGCCTTT	CCATGACGCCGTCCTTTC	GLUT^51^
H24	Glut4	Glucose transporter 4	CGTGGGCGGCATGATT	CCAGCATGGCCCTTTTCC	GLUT^51^
H25	Hif1a	Hypoxia inducible factor 1 subunit alpha	GGTGGATATGTCTGGGTTGAAAC	ATGCACTGTGGTTGAGAATTCTTG	TTP^102^
H26	Hmgr	3-Hydroxy-3-methylglutaryl-CoA reductase	AAGTGAAAGCCTGGCTCGAA	CTAGTGCTGTCAAATGCCTCCTT	^103^
H27	Hmox1	Heme oxygenase 1	CTTCTCCGATGGGTCCTTACACT	TCACATGGCATAAAGCCCTACA	TTP^104^
H28	Hua	Human antigen a	GATCCTCTGGCAGATGTTTGG	CGCGGATCACTTTCACATTG	Gossypol^40^
H29	Icam1	Intercellular adhesion molecule 1/CD54	GGAGCTTCGTGTCCTGTATGG	TTTCTGGCCACGTCCAGTTT	^105^
H30	Inos	Inducible nitric oxide synthase	AGATCCGGTTCACAGTCTTGGT	GCCATGACCTTCCGCATTAG	^106^
H31	Insr	Insulin receptor	CAACGGGCAGTTTGTCGAA	TGGTCGGGCAAACTTTCTG	^50^
H32	Il2	Interleukin 2	TATGCAGATGAGACAGCAACCAT	TTGAGATGATGCTTTGACAAAAGG	TTP^61^
H33	IL6	Interleukin 6	CCCACACAGACAGCCACTCA	CCGTCGAGGATGTACCGAAT	TTP^62^
H34	IL8	Interleukin 8	CCATCTCACTGTGTGTAAACATGACTT	ATCAGGAAGGCTGCCAAGAG	TTP^63^
H35	Il10	Interleukin 10	GCCGTGGAGCAGGTGAAG	TGGCTTTGTAGATGCCTTTCTCT	TTP^64^
H36	Il12	Interleukin 12	TGCCTTCACCACTCCCAAA	TGTCTGGCCTTCTGGAGCAT	TTP^65^
H37	Il16	Interleukin 16	CAGGGCCTCACACGGTTT	GACAATCGTGACAGGTCCATCA	TTP^47^
H38	Il17	Interleukin 17	CCCAAAAGGTCCTCAGATTACTACA	TCATTGCGGTGGAGATTCC	TTP^66^
H39	Leptin	Body fat and obesity hormone	AGGGAGACCGAGCGCTTT	CACATCCCTCACCTCCTTCAAA	^107^
H40	Map1lc3a	Microtubule-associated proteins 1 light chain 3A	GTGAACCAGCACAGCATGGT	CCTCGTCTTTCTCCTGCTCGTA	^108^
H41	Map1lc3b	Microtubule-associated proteins 1 light chain 3B	AGGCGCTTACAGCTCAATGC	ACCATGCTGTGTCCGTTCAC	^108^
H42	Nfkb	Nuclear factor kappa B	GGTGCCTCTAGTGAAAAGAACAAGA	GCTGGTCCCACATAGTTGCA	^109^
H43	P53	Tumor suppressor	CTTGCAATAGGTGTGCGTCAGA	GGAGCCCCGGGACAAA	Gossypol^33^
H44	Pim1	Proto-oncogene serine/threonine-protein kinase	TGCTCCACCGCGACATC	TGAGCTCGCCGCGATT	TTP^43^
H45	Pparr	Peroxisome proliferator-activated receptor gamma	GAACGACCAAGTAACTCTCCTCAAA	CAAGGAGGCCAGCATTGTGT	Gossypol^36^
H46	Rab24	Ras-related oncogene 24	TCGGTCGGAGACGCACTT	TGGCCTCATAGCGCTCAGA	^110^
H47	Rpl32	Ribosomal protein L32 (60S ribosomal unit)	CCTCCAAGAACCGCAAAGC	GGTGACTCTGATGGCCAGTTG	^80^
H48	Tnf	Tumor necrosis factor	GGAGAAGGGTGACCGACTCA	CAGACTCGGCAAAGTCGAGAT	TTP^62^
H49	Tnfsf10	Tumor necrosis factor superfamily, member 10	GCTCTGGGCCGCAAAAT	AGGAATGAATGCCCACTCCTT	Gossypol^39^
H50	Ulk2	Unc-51 like autophagy activating kinase 2	ACAGCTCCTTTCAAAATCCCTAAA	AGGCCCATGACGAGTAACCA	^111^
H51	Vegf	Vascular endothelial growth factor	CCCACTGAGGAGTCCAACATC	GGCCTTGGTGAGGTTTGATC	TTP^41^
H52	Zfand5	Zinc finger AN1-type containing 5	AGGGTTTGACTGCCGATGTG	ACTGGATTCTCTTTTCTGATTTTTGC	TTP^84^
H53	Zfp36	Zinc finger protein 36	GGCGACTCCCCATCTTCAA	GACCGGGCAGTCACTTTGTC	TTP^50^
H54	Zfp36L1	Zinc finger protein 36 like 1	TCTGCCACCATCTTCGACTTG	TGGGAGCACTATAGTTGAGCATCT	TTP^50^
H55	Zfp36L2	Zinc finger protein 36 like 2	CCTTTCATACCATCGGCTTCTG	TCGTCCGCGTTGTGGAT	TTP^50^

## Results

### Effect of gossypol on colon cancer cell viability

**Figure 2:**
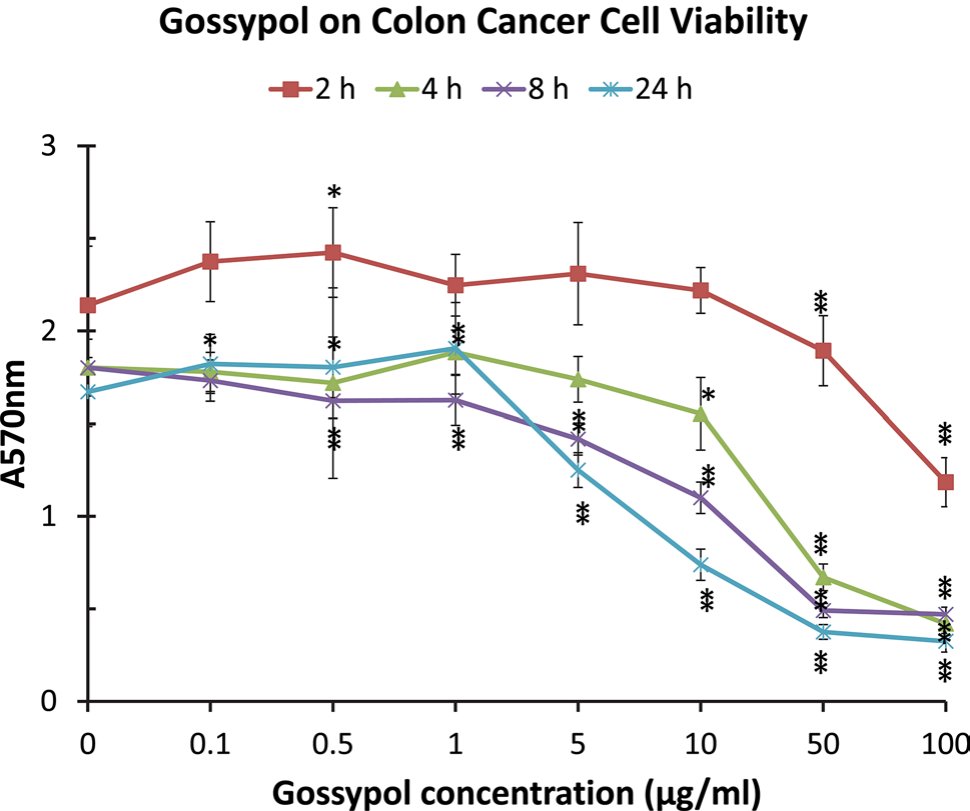
Effect of gossypol on human colon cancer cell viability. Human colon cancer cells (COLO 225) were treated with various concentrations of gossypol for 2, 4, 8 and 24 h. The cell media were added with MTT assay reagent, and incubated for 2 h before adding MTT solubilization solution, shaken at room temperature overnight. “0 µg/mL” treatment corresponding to 1% DMSO in the culture medium, the vehicle control for the experiment. The color density in the wells was recorded at A570. The data represent the mean and standard deviation of 12 independent samples. “*” and “**” near the data points represent significance between the treatment and the control at *p* < 0.05 and *p* < 0.01, respectively.

Cell cytotoxicity of human colon cancer cells (COLO 225) was determined with MTT method after the cells were treated for 2, 4, 8 and 24 h with 0.1–100 µg/mL of gossypol. MTT assay showed that gossypol significantly reduced colon cancer cell survival under high concentrations or long time treatment (Fig. 2). The cell viability was reduced to 55% of the control by 100 µg/mL gossypol after being treated for 2 h. The cell viability was reduced to a quarter of the control by 100 µg/mL gossypol after being treated for 4 or 8 h. The cell viability was further reduced to only 19% of the control by 100 µg/mL gossypol treatment for 24 h. There were clear dosage effects on reducing colon cancer cell viability after 1 µg/mL of gossypol treatment (Fig. 2).

### Basal expression level of selected genes in human colon cancer cells

To provide a basis for the comparison of gene expression regulation by gossypol in the colon cancer cells, we first measured the relative mRNA levels of 55 genes in the cells treated with 1% DMSO control for 8 h by SYBR Green qPCR assay using the specific primers (Table 1). The qPCR assay showed that the cycle of threshold (Cq) of BCL2 mRNA was one of the mRNAs with minimal variation among the 55 genes analyzed. The mean ± standard deviation of Cq for BCL2 mRNA was 29.39 ± 1.08 (n = 24) (Table 2, left column). The mRNA levels of GAPDH and RPL32 were the most abundant in the cells with 39 and 42 fold of BCL2 mRNA, respectively, whereas INOS mRNA was undetectable and AHRR1, COX1, CYCLIND1, GLUT4, ICAM1, IL10, IL12, RAB24, VEGF and ZFP36L2 mRNAs were minimally detected with less than 5% of BCL2 mRNA in the colon cancer cells (Table 2, left column).

**Table 2:** Basal level, reference mRNA and gossypol effects on 55 gene expression.

ID	mRNA	DMSO control		Gossypol		Gossypol/control
		Mean ± SD	Fold of Bcl2	Mean ± SD	Fold of Bcl2	Fold of control
H1	Ahrr1	33.88 ± 1.14	*0.04*	33.49 ± 1.09	*0.03*	0.65
**H2**	**Bcl2**	**29.39** ± **1.08**	**1.00**	**28.37** ± **1.08**	**1.00**	**1.00**
H3	Bcl2l1	27.64 ± 2.34	***3.35***	26.63 ± 1.81	***3.34***	1.00
H4	Bnip3	27.54 ± 1.22	***3.60***	26.47 ± 1.14	***3.74***	1.04
H5	Cd36	28.45 ± 1.23	1.91	27.55 ± 1.16	1.77	0.92
H6	Claudin1	28.03 ± 4.30	***2.56***	27.85 ± 3.73	1.44	0.56
H7	Cox1	34.92 ± 4.79	*0.02*	40.95 ± 5.37	*0*	*0.01*
H8	Cox2	30.65 ± 1.62	*0.42*	30.85 ± 2.80	*0.18*	*0.43*
H9	Csnk2a1	26.02 ± 1.95	***10.33***	25.22 ± 1.48	***8.90***	0.86
H10	Ctsb	28.13 ± 2.73	***2.38***	27.34 ± 2.01	***2.04***	0.86
H11	Cxcl1	32.75 ± 2.60	*0.10*	31.46 ± 1.88	*0.12*	1.22
H12	Cyclind1	34.10 ± 5.42	*0.04*	33.59 ± 3.81	*0.03*	0.70
H13	Cyp19a1	32.40 ± 3.37	*0.12*	35.65 ± 2.03	*0.01*	*0.05*
H14	Dgat1	29.38 ± 1.86	1.00	28.55 ± 1.67	0.88	0.88
H15	Dgat2a	31.80 ± 2.18	*0.19*	31.48 ± 2.30	*0.12*	0.62
H16	Dgat2b	30.88 ± 1.79	*0.35*	30.37 ± 2.56	*0.25*	0.71
H17	E2f1	29.61 ± 1.08	0.85	29.72 ± 2.56	*0.39*	0.46
H18	Elk1	30.82 ± 2.84	*0.37*	31.22 ± 1.80	*0.14*	0.38
H19	Fas	30.63 ± 4.60	*0.42*	28.97 ± 2.49	0.66	1.57
H20	Gapdh	24.10 ± 4.01	***39.09***	22.66 ± 2.95	***52.62***	1.35
H21	Glut1	27.24 ± 2.54	***4.41***	26.80 ± 2.41	***2.98***	0.67
H22	Glut2	29.22 ± 1.81	1.12	27.72 ± 1.13	1.58	1.40
H23	Glut3	28.61 ± 1.31	1.71	27.00 ± 1.60	***2.59***	1.51
H24	Glut4	41.48 ± 4.81	*0*	41.17 ± 3.30	*0*	0.61
H25	Hif1a	27.64 ± 2.33	***3.36***	26.82 ± 2.11	***2.93***	0.87
H26	Hmgr	27.50 ± 1.91	***3.70***	26.46 ± 1.47	***3.76***	1.02
H27	Hmox1	29.68 ± 1.41	0.82	28.47 ± 1.37	0.94	1.15
H28	Hua	32.25 ± 3.52	*0.14*	30.64 ± 2.34	*0.21*	1.51
H29	Icam1	33.93 ± 4.12	*0.04*	32.23 ± 1.50	*0.07*	1.61
H30	Inos	ud	*ud*	ud	*ud*	ud
H31	Insr	29.81 ± 3.12	0.74	29.58 ± 1.96	*0.43*	0.58
H32	Il2	31.27 ± 1.24	*0.27*	29.93 ± 1.19	*0.34*	1.25
H33	IL6	29.09 ± 1.39	1.23	27.29 ± 1.17	0.63	1.72
H34	IL8	29.14 ± 1.20	1.19	28.54 ± 1.52	0.89	0.75
H35	Il10	41.42 ± 5.26	*0*	39.31 ± 8.57	*0*	***2.14***
H36	Il12	37.55 ± 3.02	*0*	34.24 ± 4.17	*0.02*	***4.94***
H37	Il16	28.49 ± 1.17	1.87	28.57 ± 2.12	0.87	*0.47*
H38	Il17	29.56 ± 1.38	0.89	28.00 ± 1.30	1.29	1.46
H39	Leptin	29.51 ± 1.22	0.92	28.34 ± 0.95	1.03	1.12
H40	Map1lc3a	29.48 ± 1.87	0.94	27.84 ± 1.14	1.45	1.55
H41	Map1lc3b	26.18 ± 1.73	***9.25***	25.25 ± 1.79	***8.72***	0.94
H42	Nfkb	31.08 ± 2.97	*0.31*	30.21 ± 2.36	*0.28*	0.91
H43	P53	31.06 ± 2.58	*0.31*	30.22 ± 2.04	*0.28*	0.89
H44	Pim1	29.13 ± 1.09	1.20	28.25 ± 1.56	1.09	0.91
H45	Pparr	29.63 ± 1.50	0.85	29.69 ± 1.36	*0.40*	0.47
H46	Rab24	42.25 ± 3.40	*0*	40.54 ± 3.54	*0*	1.62
H47	Rpl32	24.00 ± 3.68	***41.78***	22.94 ± 2.94	***43.17***	1.03
H48	Tnf	30.88 ± 1.71	*0.35*	29.74 ± 1.21	*0.39*	1.10
H49	Tnfsf10	28.22 ± 1.48	***2.24***	27.72 ± 1.21	1.57	0.70
H50	Ulk2	29.20 ± 1.11	1.14	27.82 ± 1.08	1.46	1.28
H51	Vegf	40.47 ± 4.57	*0*	38.68 ± 5.16	*0*	1.71
H52	Zfand5	27.20 ± 1.50	***4.55***	26.31 ± 1.49	***4.19***	0.92
H53	Zfp36	28.86 ± 1.85	1.44	27.37 ± 1.65	***2.00***	1.39
H54	Zfp36L1	29.29 ± 3.07	1.07	28.92 ± 2.44	0.69	0.64
H55	Zfp36L2	41.60 ± 3.61	*0*	39.81 ± 3.04	*0*	1.71

The data represent the mean and standard deviation of 24 independent samples. The fold was calculated using the mean data. ud: undetected. Bold italics: Genes with mRNA levels at least twofold of Bcl2, roughly interpreted as their expression more abundant than that of Bcl2. Italics: Genes with mRNA levels less than 50% of Bcl2, roughly interpreted as their expression less abundant than that of Bcl2.

Genes with mRNA levels at least twofold of BCL2 mRNA could be roughly interpreted as their expression more abundant than that of BCL2 mRNA. There were 13 more abundantly expressed genes than BCL2 mRNA including mRNAs of BCL2L1 (3.35 fold), BNIP3 (3.60 fold), CLUADIN1 (2.56 fold), CSNK2A1 (10.33 fold), CTSB (2.38 fold), GAPDH (39.09 fold), GLUT1 (4.41 fold), HIF1A (3.36 fold), HMGR (3.70 fold), MAP1LC3B (9.25 fold), RPL32 (41.78 fold), TNFSF10 (2.24 fold) and ZFAND5 (4.55 fold) (Table 2, left column). Genes with mRNA levels less than 50% of BCL2 mRNA could be roughly interpreted as their expression less abundant than that of BCL2 mRNA. There were 22 less abundantly expressed genes including mRNAs of AHRR1 (4%), COX1 (2%), COX2 (42%), CXCL1 (10%), CYCLIND1 (4%), CYP19A1 (12%), DGAT2A (19%), DGAT2B (35%), ELK1 (37%), FAS (42%), GLUT4 (< 1%), HUA (14%), ICAM1 (4%), IL2 (27%), IL10 (< 1%), IL12 (< 1%), NFKB (31%), P53 (31%), RAB24 (< 1%), TNF (35%), VEGF (< 1%) and ZFP36L2 (< 1%) (Table 2, left column). TaqMan qPCR assay showed similar trend as of SYBR Green qPCR (data not shown). SYBR Green qPCR assay was chosen to conduct gene expression analysis in the following experiments for cost saving and convenience.

### Selection of reference gene for qPCR assays in human colon cancer cells

Reference gene for qPCR assays should be stably expressed without much variation by the experimental treatments. The less of standard deviation of Cq among the gossypol treatments might be indication of the more stable expression of the gene which could be served as an internal reference. To identify which gene could be served as an internal reference in the human colon cancer cells in our study, we pooled the data from triplicate each of the 8 concentrations (0, 0.1, 0.5, 1, 5, 10, 50 and 100 µg/mL of gossypol) and calculated the mean ± standard deviation from the 24 samples (Table 2, middle column). The Cq value of BCL2 mRNA was among the least varied in the cells with the mean ± standard deviation of 28.37 ± 1.08 (n = 24) (Table 2, middle column). The Cq value of other mRNAs with similar standard deviations were mRNAs of AHRR1 (1.09), BNIP3 (1.14), CD36 (1.16), GLUT2 (1.13), IL2 (1.19), IL6 (1.17), LEPTIN (0.95), MAP1LC3A (1.14) and ULK2 (1.08). GAPDH and RPL32 mRNAs were widely used as references for qPCR assays in mammalian cells. However, GAPDH and RPL32 genes were shown here with much larger standard deviations (2.95 and 2.94, respectively). Furthermore, GAPDH and RPL32 mRNAs were the most abundantly expressed in the cells with 53 and 43 fold of BCL2 mRNA, respectively, which were tens of fold higher than almost all of the other mRNAs analyzed in this study (Table 2, middle column). The large standard deviations and high expression levels of GAPDH and RPL32 mRNAs suggest that they were not good internal reference genes for qPCR assays in the human colon cancer cell. Since BCL2 was widely studied in colon cancer cells and was among the least regulated genes by gossypol, we therefore selected BCL2 mRNA as the internal reference for our qPCR analyses.

There were 13 genes with mRNA levels at least twofold of BCL2 mRNA in the 24 pooled samples including mRNAs of BCL2L1 (3.34 fold), BNIP3 (3.74 fold), CSNK2A1 (8.90 fold), CTSB (2.04 fold), GAPDH (52.62 fold), GLUT1 (2.98 fold), GLUT3 (2.59 fold), HIF1A (2.93 fold), HMGR (3.76 fold), MAP1LC3B (8.72 fold), RPL32 (43.17 fold), ZFAND5 (4.19 fold) and ZFP36 (twofold) (Table 2, middle column). These gene expression patterns from pooled gossypol samples were similar to those from DMSO control samples except that CLAUDIN1 and TNFSF10 were expressed more in DMSO samples but GLUT3 and ZFP36 were expressed more in the gossypol pooled samples (Table 2, left vs. middle columns). There were 24 genes with mRNA levels less than 50% of BCL2 mRNA including mRNAs of AHRR1 (3%), COX1 (< 1%), COX2 (18%), CXCL1 (12%), CLAUDIN1 (3%), CYP19A1 (1%), DGAT2A (12%), DGAT2B (25%), E2F1 (39%), ELK1 (14%), GLUT4 (< 1%), HUA (21%), ICAM1 (7%), INSR (43%), IL2 (34%), IL10 (< 1%), IL12 (2%), NFKB (28%), P53 (28%), PPARR (40%), RAB24 (< 1%), TNF (39%), VEGF (< 1%) and ZFP36L2 (< 1%) (Table 2, middle column). These gene expression patterns from gossypol pooled samples were similar to those from DMSO control samples except that FAS mRNA was expressed more in DMSO samples but E2F1, INSR and PPARR mRNAs were expressed more in the gossypol pooled samples (Table 2, left vs. middle columns).

### Overall effect of gossypol on gene expression in human colon cancer cells

To provide a general idea how these genes were regulated by gossypol, we analyzed the pooled qPCR data using BCL2 mRNA as the internal reference and DMSO treatment as the sample control. As shown in the right column of Table 2, expression of a number of genes was affected by gossypol. Gossypol decreased the expression of six mRNAs with less than 50% of the control and only up-regulated the expression of two mRNAs with at least twofold of the control. The up-regulated mRNAs were IL10 (2.14 fold) and IL12 (4.94 fold) (Table 2, right column). The down-regulated mRNAs were COX1 (1%), COX2 (43%), CYP19A1 (5%), E2F1 (46%), ELK1 (38%) and PPARR (47%) (Table 2, right column).

### Dosage effect of gossypol on gene expression in human colon cancer cells

Human colon cancer cells were treated with 8 concentrations of gossypol (0, 0.1, 0.5, 1, 5, 10, 50 and 100 µg/mL of gossypol). SYBR Green qPCR analyzed the expression of all 55 genes with BCL2 mRNA as an internal reference and 1% DMSO treatment as the sample control (Table 3). Generally, most of the gene expression was suppressed by higher concentrations of gossypol (Table 3). These genes include BCL2L1, CLAUDIN1, CSNK2A1, CTSB, CXCL1, DGAT1, DGAT2A, DGAT2B, ELK1, FAS, GAPDH, GLUT1, HMGR, HUA, ICAM1, MAP1LC3B, NFKB, P53, RPL32, VEGF, ZFAND5, ZFP36L1 and ZFP36L2. Some of the gene expression was increased by gossypol including mRNAs of COX1, COX2, GLUT3, GLUT4, PPARR and RAB24 (Table 3). Eight of the *p* values were less than 5% threshold as shown with a “*” in the right column of Table 3, suggesting their expression levels might be statistically significant. Among them, gossypol significantly regulated the expression of genes coding for mRNAs of CLAUDIN1, ELK1, FAS, GAPDH, GGLUT3, IL2, IL8 and ZFAND5; all down-regulated except GLUT3 mRNA (Table 3, highlighted row). TTP family mRNA levels (ZFP36, ZFP36L1 and ZFP36L2 mRNAs) were generally decreased and TTP-targeted cytokine mRNA levels (TNF, COX2, PPARR and RAB24 mRNAs) were relatively high in the colon cancer cells (Table 3). More specific analyses of gene expression under gossypol treatments are described below according to specific gene families.

**Table 3:** Gossypol Dosages on Colon Cancer Cell Gene Expression.

ID	mRNA	DMSO	0.1 µg/mL	0.5 µg/mL	1 µg/mL	5 µg/mL	10 µg/mL	50 µg/mL	100 µg/mL	*p* value
		Fold	Mean ± SD	Mean ± SD	Mean ± SD	Mean ± SD	Mean ± SD	Mean ± SD	Mean ± SD	
H1	Ahrr1	1.00	3.54 ± 2.16	2.91 ± 0.00	1.33 ± 0.51	1.41 ± 1.91	0.50 ± 0.17	1.51 ± 9.00	ud	0.226
**H2**	**Bcl2**	**1.00**	**1.00**	**1.00**	**1.00**	**1.00**	**1.00**	**1.00**	**1.00**	
H3	Bcl2l1	1.00	1.29 ± 1.15	0.82 ± 0.28	0.82 ± 0.13	1.19 ± 1.24	0.33 ± 0.08	0.34 ± 0.06	0.25 ± 0.06	0.015
H4	Bnip3	1.00	0.91 ± 0.37	1.27 ± 0.72	0.80 ± 0.20	1.08 ± 0.45	0.55 ± 0.10	1.03 ± 0.14	0.55 ± 0.15	0.200
H5	Cd36	1.00	0.87 ± 0.11	1.12 ± 0.35	0.79 ± 0.42	1.36 ± 0.56	0.91 ± 0.20	1.09 ± 0.46	0.64 ± 0.12	0.304
***H6***	***Claudin1***	***1.00 a***	***1.72 ± 2.30 a***	***0.64 ± 0.19 a***	***0.77 ± 0.33 a***	***0.77 ± 0.88 a***	***0.20 ± 0.14 ab***	***0.09 ± 0.11 ab***	***0.005 ± 0.003 b***	0.03*
H7	Cox1	1.00	2.08 ± 1.86	0.30 ± 0.44	1.15 ± 0.98	1.23 ± 2.08	1.35 ± 1.14	1.28 ± 1.11	6.60 ± 5.96	0.748
H8	Cox2	1.00	1.87 ± 2.90	2.35 ± 1.69	1.76 ± 2.13	3.69 ± 0.39	0.97 ± 1.22	3.61 ± 4.60	0.55 ± 0.47	0.370
H9	Csnk2a1	1.00	1.00 ± 0.68	0.70 ± 0.13	0.67 ± 0.18	0.87 ± 0.40	0.38 ± 0.14	0.33 ± 0.09	0.25 ± 0.07	0.032
H10	Ctsb	1.00	1.47 ± 1.02	0.95 ± 0.26	0.84 ± 0.31	0.90 ± 1.09	0.24 ± 0.14	0.20 ± 0.06	0.14 ± 0.07	0.029
H11	Cxcl1	1.00	1.66 ± 0.74	1.45 ± 0.42	1.11 ± 0.55	1.53 ± 0.22	0.05 ± 0.08	0.03 ± 0.04	0.0001 ± 0.000	0.043
H12	Cyclind1	1.00	ud	ud	ud	ud	ud	ud	ud	
H13	Cyp19a1	1.00	0.77 ± 0.38	0.07 ± 0.07	19.49 ± 32.77	10.07 ± 14.2	0.44 ± 0.62	0.71 ± 1.15	3.17 ± 3.27	0.509
H14	Dgat1	1.00	0.78 ± 0.67	0.34 ± 0.08	0.53 ± 0.07	0.73 ± 0.49	0.31 ± 0.07	0.38 ± 0.16	0.19 ± 0.06	0.073
H15	Dgat2a	1.00	1.37 ± 1.24	1.20 ± 0.62	0.06 ± 0.04	1.23 ± 0.82	0.40 ± 0.36	0.48 ± 0.09	0.11 ± 0.08	0.089
H16	Dgat2b	1.00	0.08 ± 0.08	1.05 ± 0.53	0.43 ± 0.07	0.8 ± 0.06	0.57 ± 0.49	0.35 ± 0.33	0.28 ± 0.22	0.036
H17	E2f1	1.00	0.47 ± 0.44	0.39 ± 0.56	1.11 ± 0.65	1.64 ± 0.41	0.85 ± 0.75	1.44 ± 0.34	1.03 ± 0.10	0.075
***H18***	***Elk1***	***1.00 a***	***0.22 ± 0.11 b***	***0.15 ± 0.06 b***	***0.12 ± 0.07 b***	***0.08 ± 0.07 b***	***0.06 ± 0.03 b***	***0.06 ± 0.05 b***	***0.01 ± 0.00 b***	0.001*
***H19***	***Fas***	***1.00 a***	***1.25 ± 1.00 a***	***0.44 ± 0.06 ab***	***0.66 ± 0.25 ab***	***0.50 ± 0.47 ab***	***0.11 ± 0.07 b***	***0.10 ± 0.13 b***	***0.01 ± 0.01 c***	0.008*
***H20***	***Gapdh***	***1.00 a***	***1.09 ± 1.05 a***	***0.48 ± 0.36 a***	***0.69 ± 0.13 a***	***0.63 ± 0.70 a***	***0.26 ± 0.15 a***	***0.17 ± 0.21 a***	***0.03 ± 0.01 b***	0.046*
H21	Glut1	1.00	0.94 ± 0.53	0.56 ± 0.22	0.74 ± 0.75	0.65 ± 1.04	0.98 ± 0.31	0.39 ± 0.31	0.11 ± 0.07	0.269
H22	Glut2	1.00	0.84 ± 0.23	1.14 ± 0.59	0.84 ± 0.27	1.37 ± 0.51	0.83 ± 0.09	1.02 ± 0.20	0.58 ± 0.14	0.180
*H23*	*Glut3*	*1.00 b*	*5.48* ± *2.75 ab*	*5.75* ± *0.73 ab*	*5.80* ± *2.90 ab*	*3.22* ± *1.37 ab*	*6.78* ± *0.46 a*	*6.37* ± *1.95 a*	*4.34* ± *2.10 ab*	0.023*
H24	Glut4	1.00	2.77 ± 3.11	2.83 ± 1.04	31.14 ± 41.70	14.86 ± 17.21	5.75 ± 4.75	12.26 ± 16.28	3.70 ± 5.35	0.507
H25	Hif1a	1.00	3.63 ± 4.39	1.89 ± 0.60	1.75 ± 0.59	1.09 ± 0.89	0.82 ± 0.29	0.64 ± 0.15	0.34 ± 0.14	0.039
H26	Hmgr	1.00	0.93 ± 0.82	0.62 ± 0.31	0.58 ± 0.21	0.51 ± 0.26	0.32 ± 0.06	0.33 ± 0.14	0.38 ± 0.41	0.189
H27	Hmox1	1.00	0.66 ± 0.33	0.63 ± 0.11	1.15 ± 1.09	0.72 ± 0.11	0.50 ± 0.11	0.63 ± 0.16	0.35 ± 0.02	0.096
H28	Hua	1.00	1.81 ± 1.45	0.98 ± 0.34	0.71 ± 0.31	0.63 ± 0.45	0.39 ± 0.17	0.30 ± 0.19	0.07 ± 0.06	0.032
H29	Icam1	1.00	0.70 ± 0.34	0.33 ± 0.14	0.54 ± 0.16	0.48 ± 0.44	0.13 ± 0.06	0.07 ± 0.07	0.06 ± 0.00	0.091
H30	Inos	ud	ud	ud	ud	ud	ud	ud	ud	
H31	Insr	1.00	2.51 ± 1.84	1.13 ± 0.48	1.38 ± 0.82	0.72 ± 0.75	0.34 ± 0.29	0.52 ± 0.42	0.05 ± 0.00	0.113
***H32***	***Il2***	***1.00 ab***	***0.95 ± 0.08 ab***	***1.49 ± 0.29 a***	***0.98 ± 0.13 ab***	***1.040.49 ab***	***0.83 ± 0.33 ab***	***0.92 ± 0.18 ab***	***0.60 ± 0.20 b***	0.04*
H33	IL6	1.00	1.50 ± 0.43	1.79 ± 1.08	1.72 ± 1.00	1.81 ± 0.59	1.47 ± 0.48	1.49 ± 0.44	1.00 ± 0.37	0.623
***H34***	***IL8***	***1.00 ab***	***1.05 ± 0.44 a***	***1.22 ± 0.19 a***	***0.72 ± 0.17 ab***	***1.23 ± 0.42 a***	***0.88 ± 0.14 ab***	***0.94 ± 0.08 ab***	***0.36 ± 0.22 b***	0.013*
H35	Il10	1.00	ud	ud	ud	ud	ud	ud	ud	
H36	Il12	1.00	ud	ud	ud	ud	ud	ud	ud	
H37	Il16	1.00	0.003 ± 0.001	0.003 ± 0.001	0.003 ± 0.001	0.001 ± 0.001	0.00	0.002 ± 0.001	0.001 ± 0.001	
H38	Il17	1.00	0.86 ± 0.49	1.87 ± 1.03	1.11 ± 0.33	1.91 ± 0.71	1.04 ± 0.53	1.54 ± 0.46	0.93 ± 0.37	0.179
H39	Leptin	1.00	0.74 ± 0.21	1.15 ± 0.50	1.25 ± 0.10	1.18 ± 0.51	0.95 ± 0.25	1.08 ± 0.24	1.17 ± 0.35	0.604
H40	Map1lc3a	1.00	0.94 ± 0.55	1.05 ± 0.47	1.01 ± 0.45	0.90 ± 0.31	0.57 ± 0.06	0.61 ± 0.05	0.60 ± 0.09	0.136
H41	Map1lc3b	1.00	0.91 ± 0.98	0.57 ± 0.19	0.94 ± 0.68	0.68 ± 0.47	0.33 ± 0.03	0.31 ± 0.20	0.19 ± 0.09	0.132
H42	Nfkb	1.00	0.96 ± 1.11	0.52 ± 0.18	0.47 ± 0.10	0.44 ± 0.51	0.20 ± 0.16	0.17 ± 0.21	0.02 ± 0.00	0.151
H43	P53	1.00	1.45 ± 1.30	0.29 ± 0.25	0.64 ± 0.29	0.73 ± 0.53	0.24 ± 0.20	0.25 ± 0.14	0.22 ± 0.06	0.128
H44	Pim1	1.00	0.69 ± 0.15	0.68 ± 0.33	0.65 ± 0.30	0.69 ± 0.63	0.69 ± 0.37	1.01 ± 0.10	0.62 ± 0.12	0.616
H45	Pparr	1.00	1.39 ± 0.97	2.00 ± 0.91	2.72 ± 2.49	2.57 ± 1.34	1.61 ± 0.68	1.49 ± 1.13	9.39 ± 12.65	0.731
H46	Rab24	1.00	25.49 ± 31.18	10.76 ± 10.86	1.68 ± 0.00	1.36 ± 1.82	8.68 ± 12.21	1.69 ± 1.14	6.41 ± 6.64	0.630
H47	Rpl32	1.00	1.06 ± 0.90	0.68 ± 0.43	0.68 ± 0.22	0.46 ± 0.45	0.19 ± 0.11	0.15 ± 1.18	0.02 ± 0.01	0.029
H48	Tnf	1.00	1.22 ± 0.57	1.24 ± 0.32	1.16 ± 0.52	1.47 ± 0.38	0.85 ± 0.29	1.29 ± 0.14	0.71 ± 0.23	0.241
H49	Tnfsf10	1.00	0.94 ± 0.30	1.23 ± 0.76	0.93 ± 0.39	1.26 ± 0.32	0.57 ± 0.57	1.01 ± 0.15	0.58 ± 0.14	0.342
H50	Ulk2	1.00	0.88 ± 0.12	1.61 ± 0.79	0.99 ± 0.37	1.22 ± 0.34	1.50 ± 0.56	1.19 ± 0.35	0.84 ± 0.15	0.273
H51	Vegf	1.00	0.001 ± 0.001	0.01 ± 0.02	0.26 ± 0.35	0.001 ± 0.000	0.00002 ± 0.00002	0.0001 ± 0.0001	0.05 ± 0.00	0.107
***H52***	***Zfand5***	***1.00 ab***	***0.61 ± 0.47 a***	***0.54 ± 0.21 ab***	***0.53 ± 0.04 ab***	***0.51 ± 0.17 ab***	***0.35 ± 0.05 ab***	***0.39 ± 0.12 ab***	***0.20 ± 0.10 b***	0.048*
H53	Zfp36	1.00	1.00 ± 0.75	0.65 ± 0.20	0.59 ± 0.08	0.72 ± 0.51	0.70 ± 0.11	0.81 ± 0.64	0.24 ± 0.08	0.243
H54	Zfp36L1	1.00	1.60 ± 1.48	0.12 ± 0.10	0.77 ± 0.07	0.83 ± 0.77	0.13 ± 0.09	0.07 ± 0.04	0.02 ± 0.00	0.050
H55	Zfp36L2	1.00	0.32 ± 0.42	0.90 ± 0.89	0.74 ± 0.97	0.08 ± 0.00	0.15 ± 0.15	0.18 ± 0.08	0.01 ± 0.00	0.112

The data represent the mean and standard deviation of three independent samples. Data with different lowcase letters represent significance between the treatments *p* < 0.05. ud: undetected. Bold italics: mRNA levels were statistically decreased by gossypol among the treatments with various concentrations. Italics: mRNA levels were statistically increased by gossypol among the treatments with various concentrations. "*" under P value column represents mRNA levels significantly affected by gossypol. Statistical analyses were conducted with Student–Newman–Keuls method for all pairwise multiple comparison. All pairwise multiple comparison was also performed with Tukey test yielding similar results (data not shown).

### Gossypol decreased the expression of reference GAPDH and RPL32 genes in human colon cancer cells

**Figure 3:**
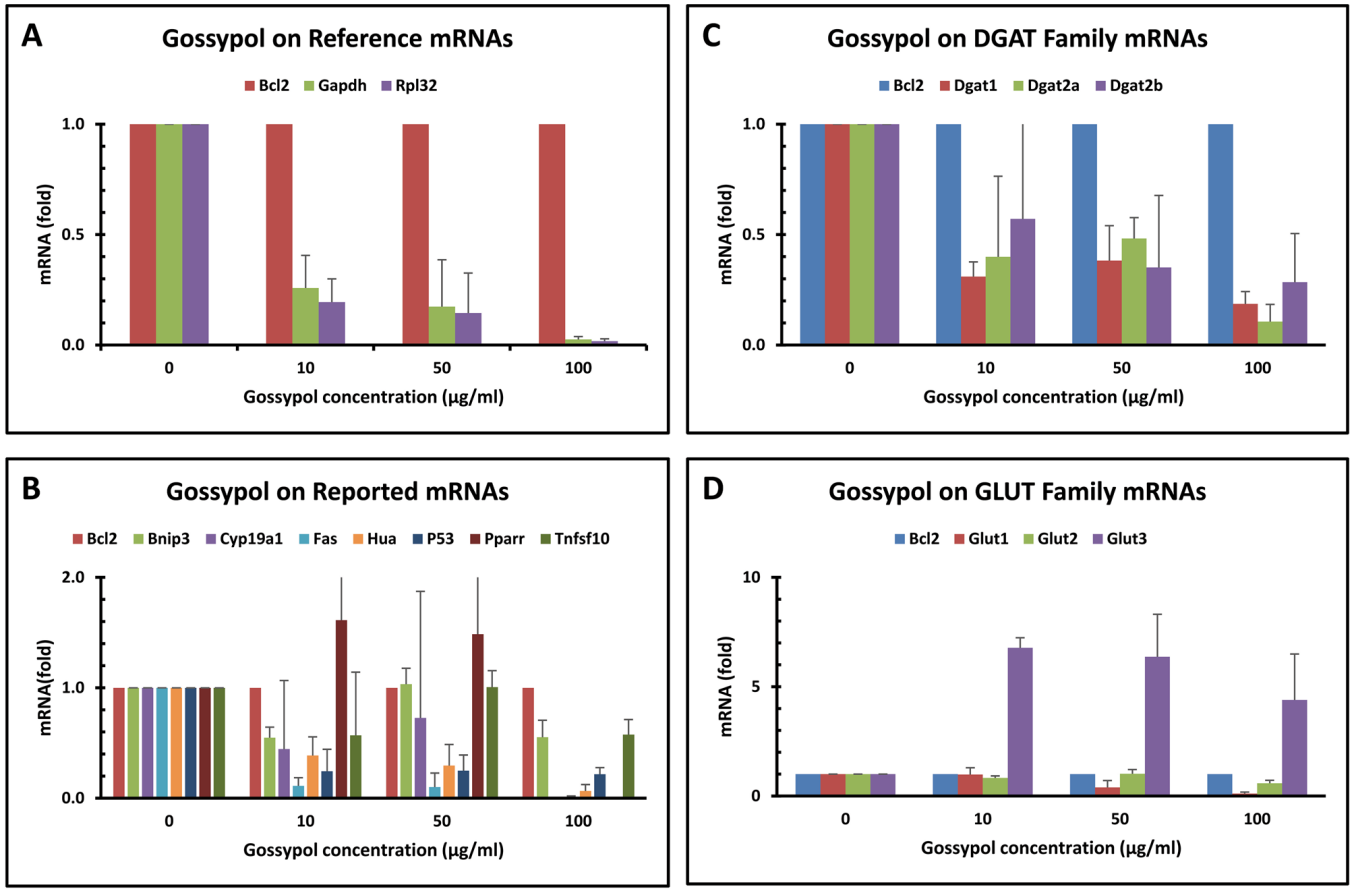
Gossypol regulated the expression of genes coded for qPCR reference mRNAs, genes reported to be regulated by gossypol, and genes coded for DGAT and GLUT mRNAs in human colon cancer cells. Human colon cancer cells (COLO 225) were treated with gossypol for 8 h. The data represent the mean and standard deviation of three independent samples. (**A**) genes coded for qPCR reference mRNAs, (**B**) genes reported to be regulated by gossypol, (**C**) genes coded for DGAT mRNAs, (**D**) genes coded for GLUT mRNAs.

The expression of the two well-known reference genes was analyzed in the colon cancer cell line under treatment with various concentration of gossypol using internal reference gene BCL2 selected in this study. The qPCR data showed that GAPDH and RPL32 mRNA levels were 39 and 42 fold of BCL2 in the controlled cells (Table 4). High concentrations of gossypol treatment resulted in a remarkable reduction of both GAPDH and RPL32 mRNA levels in the cells (Fig. 3A). Both GAPDH and RPL32 mRNA levels were reduced more than 80% by 40–100 µg/mL of gossypol treatment (Fig. 3A).

**Table 4:** Relative mRNA levels of respective gene families in the human colon cancer cells.

ID	mRNA	Mean ± SD (n = 24)	Fold
	Reference gene	Mean ± SD	Fold of Bcl2
H2	Bcl2	29.39 ± 1.08	1.00
H20	Gapdh	24.10 ± 4.01	39.09
H47	Rpl32	24.00 ± 3.68	41.78

ID	Reported gene	Mean ± SD	Fold of Bcl2
H2	Bcl2	29.39 ± 1.08	1.00
H4	Bnip3	27.54 ± 1.22	3.60
H12	Cyclind1	34.10 ± 5.42	0.04
H13	Cyp19a1	32.40 ± 3.37	0.12
H19	Fas	30.63 ± 4.60	0.42
H28	Hua	32.25 ± 3.52	0.14
H43	P53	31.06 ± 2.58	0.31
H45	Pparr	29.63 ± 1.50	0.85
H49	Tnfsf10	28.22 ± 1.48	2.24

ID	DGAT family	Mean ± SD	Fold of Dgat1
H14	Dgat1	29.38 ± 1.86	1.00
H15	Dgat2a	31.80 ± 2.18	0.19
H16	Dgat2b	30.88 ± 1.79	0.35

ID	GLUT family	Mean ± SD	Fold of Glut1
H21	Glut1	27.24 ± 2.54	1.00
H22	Glut2	29.22 ± 1.81	0.25
H23	Glut3	28.61 ± 1.31	0.39
H24	Glut4	41.48 ± 4.81	0.00

ID	TTP family	Mean ± SD	Fold of Zfp36
H53	Zfp36	28.86 ± 1.85	1.00
H54	Zfp36L1	29.29 ± 3.07	0.74
H55	Zfp36L2	41.60 ± 3.61	0.00

ID	IL family	Mean ± SD	Fold of Zfp36
H53	Zfp36	28.86 ± 1.85	1.00
H32	Il2	31.27 ± 1.24	0.19
H33	IL6	29.09 ± 1.39	0.85
H34	IL8	29.14 ± 1.20	0.83
H35	Il10	41.42 ± 5.26	0.00
H36	Il12	37.55 ± 3.02	0.00
H37	Il16	28.49 ± 1.17	1.30
H38	Il17	29.56 ± 1.38	0.62

ID	Proinflammatory gene	Mean ± SD	Fold of Zfp36
H53	Zfp36	28.86 ± 1.85	1.00
H7	Cox1	34.92 ± 4.79	0.01
H8	Cox2	30.65 ± 1.62	0.29
H28	Hua	32.25 ± 3.52	0.10
H39	Leptin	29.51 ± 1.22	0.64
H48	Tnf	30.88 ± 1.71	0.24
H49	Tnfsf10	28.22 ± 1.48	1.56
H51	Vegf	40.47 ± 4.57	0.00

ID	TTP targeted other gene	Mean ± SD	Fold of Zfp36
H53	Zfp36	28.86 ± 1.85	1.00
H1	Ahrr1	33.88 ± 1.14	0.03
H3	Bcl2l1	27.64 ± 2.34	2.33
H5	Cd36	28.45 ± 1.23	1.33
H6	Claudin1	28.03 ± 4.30	1.78
H9	Csnk2a1	26.02 ± 1.95	7.17
H10	Ctsb	28.13 ± 2.73	1.65
H11	Cxcl1	32.75 ± 2.60	0.07
H17	E2f1	29.61 ± 1.08	0.59
H18	Elk1	30.82 ± 2.84	0.26
H25	Hif1a	27.64 ± 2.33	2.33
H27	Hmox1	29.68 ± 1.41	0.57
H29	Icam1	33.93 ± 4.12	0.03
H44	Pim1	29.13 ± 1.09	0.83
H52	Zfand5	27.20 ± 1.50	3.16

ID	Other gene	Mean ± SD	Fold of Zfp36
H53	Zfp36	28.86 ± 1.85	1.00
H26	Hmgr	27.50 ± 1.91	2.57
H30	Inos	ud	ud
H31	Insr	29.81 ± 3.12	0.51
H40	Map1lc3a	29.48 ± 1.87	0.65
H41	Map1lc3b	26.18 ± 1.73	6.42
H42	Nfkb	31.08 ± 2.97	0.22
H46	Rab24	42.25 ± 3.40	0.00
H50	Ulk2	29.20 ± 1.11	0.79

The data represent the mean and standard deviation of 24 independent samples. ud: undetected. The relative mRNA levels were calculated using mean data of Bcl2 as the reference mRNA and the first mRNA in the respective gene family as the control.

### Gossypol effect on reported gene expression in human colon cancer cells

The expression of a number of genes was shown previously to be regulated by gossypol in cancer cells^20,33–39^ and macrophages^40^. We analyzed the expression of BNIP3, CYP19A1, FAS, HUA, P53, PPARR and TNFSF10 genes under various concentrations of gossypol in the colon cancer cell line using BCL2 as the internal reference gene. In general, this group of genes was expressed lower than BCL2 control except BINP3 and TNFSF10 (Table 4). The expression of all these genes except PPARR gene was inhibited to a large extent by the highest concentration of gossypol tested at 100 µg/mL (Fig. 3B). It appears that PPARR gene expression was increased but the large standard deviation among the measurements prevented from making such a conclusion (Fig. 3B).

### Gossypol effect on DGAT gene expression in human colon cancer cells

Diacylglycerol acyltransferases (DGATs) esterify *sn*-1,2-diacylglycerol with a long-chain fatty acyl-CoA and catalyze the rate-limiting step of triacylglycerol biosynthesis in eukaryotic organisms^52^. DGATs are divided into DGAT1 and DGAT2 subfamilies in animals and DGAT3 subfamily are present in plants^52–55^. DGAT2 mRNA was the major form of DGAT mRNAs in the mouse adipocytes and macrophages^56,57^. Gossypol was shown to be a strong stimulator of DGAT2 gene expression in mouse macrophages^56^. The qPCR data showed here that DGAT1 mRNA was the major form and the two variants of DGAT2 mRNA accounted for only half of the DGAT1 mRNA levels in the human colon cancer cells (Table 4). In contrast to mouse macrophages, we showed here that gossypol inhibited DGAT1 and DGAT2 expression in the human colon cancer cells (Fig. 3C).

### Gossypol effect on GLUT gene expression in human colon cancer cells

Glucose transporter (GLUT) family proteins consist of four isoforms which are responsible for glucose uptake in mammalian cells. GLUT1 mRNA is the major form and GLUT2 mRNA is undetectable in macrophages by TaqMan qPCR^51,58^. In this study, GLUT1 mRNA was also shown to be the major form of GLUT mRNAs but GLUT4 mRNA was barely detected in the colon cancer cells (Table 4). Gossypol treatment at high concentrations significantly decreased GLUT1 mRNA levels but increased GLLUT3 mRNA levels (Fig. 3D).

### Gossypol effect on TTP gene expression in human colon cancer cells

**Figure 4:**
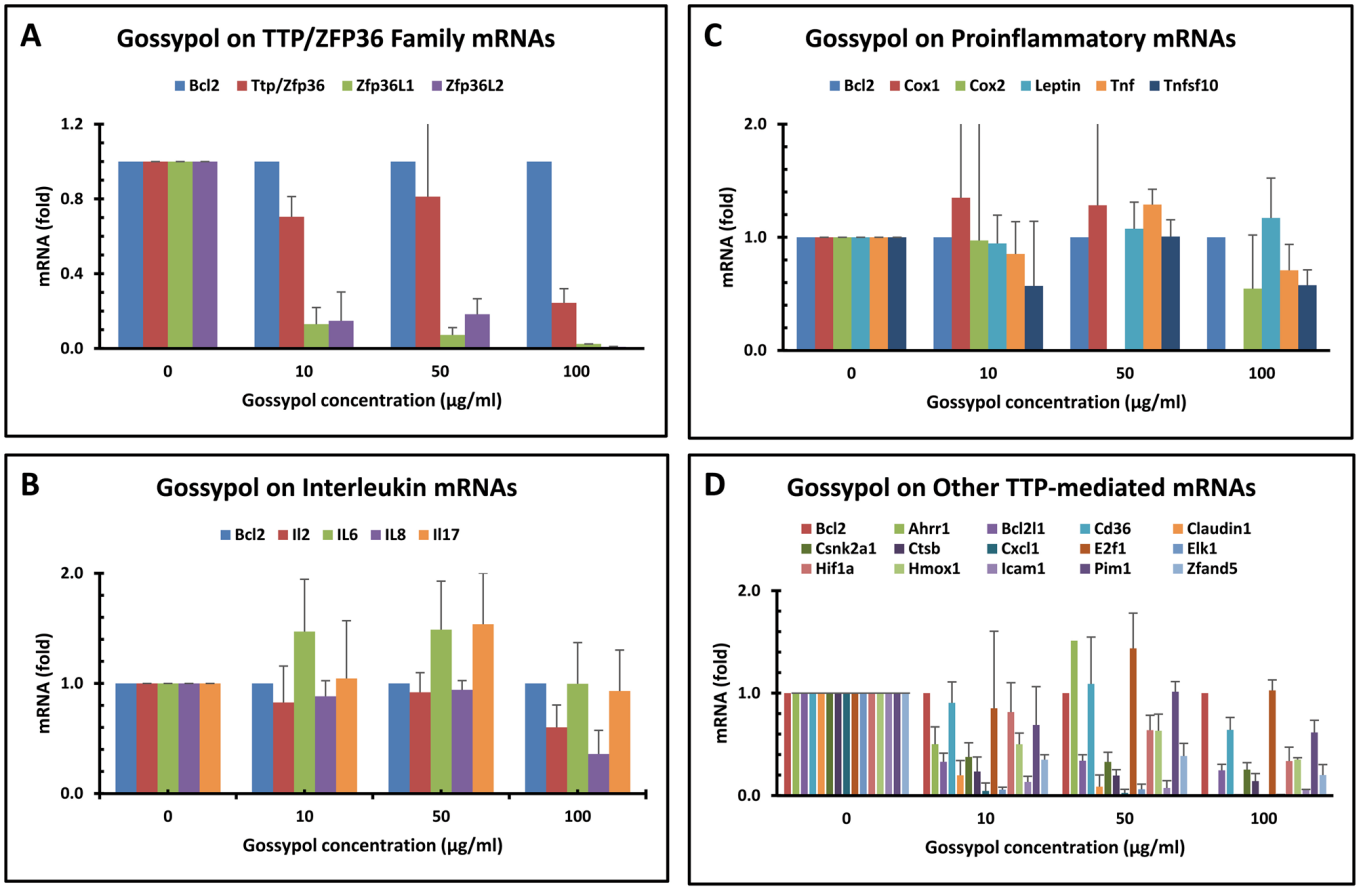
Gossypol regulated the expression of genes coded for TTP family, IL family, TTP-mediated proinflammatory cytokine and other mRNAs in human colon cancer cells. Human colon cancer cells (COLO 225) were treated with gossypol for 8 h. The data represent the mean and standard deviation of three independent samples. (**A**) genes coded for TTP family mRNAs. (**B**) genes coded for IL family mRNAs. (**C**) genes coded for TTP-mediated proinflammatory cytokine mRNAs. (**D**) genes coded for other TTP-mediated mRNAs.

Tristetraprolin (TTP/ZFP36/TIS11/G0S24/NUP475) family proteins are post-transcriptional factors controlling cytokine mRNA stability. TTP family proteins exhibit anti-inflammation effects with the potential for controlling inflammation-related diseases. TTP family proteins have three members in mammals (ZFP36 or TTP, ZFP36L1 or TIS11B, and ZFP36L2 or TIS11D) and fourth member in mouse and rat (ZFP36L3)^59,60^. SYBR Green qPCR showed that TTP/ZFP36 and ZFP36L1 were expressed in similar levels but ZFP36L2 mRNA was barely detectable in the colon cancer cells (Table 4). ZFP36, ZFP36L1 and ZFP36L2 mRNAs were all significantly reduced by high-dose of gossypol treatments (Fig. 4A).

### Gossypol effect on IL gene expression in human colon cancer cells

Several members of the interleukins (ILs) are regulated by TTP family proteins which bind to AU-rich elements (ARE) in some cytokine mRNAs and destabilizes those transcripts. TTP-mediated ILs include IL2^61^, IL6^62^, IL8^63^, IL10^64^, IL12^65^, IL16^47^ and IL17^66^. SYBR Green qPCR showed that IL10 and IL12 mRNAs were barely expressed and IL2 mRNA was low, whereas TTP and the other ILs were expressed in similar levels, which were several fold higher than IL2 mRNA in the human colon cancer cells (Table 4). The qPCR assays showed that gossypol decreased IL2 and IL8 mRNA levels but its effect on IL6 and IL17 was not apparent (Table 3 and Fig. 4B).

### Gossypol effect on proinflammatory gene expression in human colon cancer cells

The major mRNAs destabilized by TTP family proteins are proinflammatory cytokine mRNA molecules. They down-regulate the expression of mRNAs encoding cytokines such as tumor necrosis factor-alpha (TNFα)^67–70^, granulocyte–macrophage colony-stimulating factor/colony-stimulating factor 2 (GM-CSF/CSF2)^71,72^ and cyclooxygenase 2/prostaglandin-endoperoxide synthase 2 (COX2/PTGS2)^48^. TNFα and GM-CSF mRNAs are stabilized in TTP knockout mice cells^69,71^. TTP knockout mice over-express these proinflammatory cytokines which cause a severe systemic inflammatory syndrome^73,74^. TTP over-expression reduces inflammatory responses^75^. These previous studies suggest that TTP is an anti-inflammatory protein. Except TNFSF10 mRNA, all the other proinflammatory mRNAs were expressed much lower than that of TTP and VEGF mRNA was almost undetectable in the colon cancer cells (Table 4). Gossypol did not exhibit significant effects on the mRNA levels of all these proinflammatory gene expression in the human colon cancer cells (Fig. 4C).

### Gossypol effect on TTP-targeted other gene expression in human colon cancer cells

A number of other TTP-mediated mRNAs have been reported in the literature (Table 1). The basal levels of these mRNAs were either higher than that of TTP mRNA (BCL2L1, CsnK2A1, HIF1a and ZFAND5) or lower than that of TTP mRNA (Ahrr1, CXCL1, E2F1, ELK1, HMOX1 and ICAM1) (Table 4). Gossypol treatment resulted in a reduction of many of the mRNAs in the colon cancer cells (Fig. 4D).

## Discussion

Cottonseed accounts for approximately 20% of the crop value. One way to increase the value of cottonseed is to isolate bioactive compounds aimed to improving nutrition and preventing diseases. The presence of toxic polyphenolic compound gossypol in the seeds limits its use as food and feed source for humans and non-ruminant animals^2–6^. On the other hand, recent studies have shown that gossypol has potential biomedical applications. This may significantly increase cottonseed value by using gossypol from the seeds as a health intervention agent. However, it is necessary to insure safety and effectiveness of gossypol as well as the underlining molecular mechanisms before human consumption. Therefore, we evaluated the effects of gossypol on toxicity and gene expression in human colon cancer cells.

In this study, we observed that gossypol significantly decreased the cell viability of human colon cancer cells (Fig. 2). Our previous study showed that gossypol inhibited breast and pancreas cancer cell viability^76^. The effect of gossypol on decreasing breast cancer cell (MCF-7) viability^76^ was in agreement with those using gossypol, gossypol derivative, and gossypol-enriched cottonseed oil^16,17^. Gossypol also decreased pancreatic cancer cell viability after short-term treatment^76^ which is in agreement with published reports^20,77–79^.

Before we examined the effect of gossypol on gene expression in human colon cancer cells, we evaluated the relative expression levels of 55 genes and selected the internal reference for qPCR analysis since it is important for normalization of gene expression levels^80–83^. Our study showed that BCL2 mRNA was the most stable among the mRNAs from 55 genes analyzed in human colon cancer cells treated with DMSO vehicle or various concentrations of gossypol (Table 2). This result is in consistent with a previous report showing that the effect of gossypol analog on BCL2 gene expression was minimal at the mRNA level^31^. Our results showed that GAPDH and RPL32 (60S ribosomal protein 32) mRNAs were not good qPCR assay references for the colon cancer cells since they were most abundant mRNAs with large variations under the cell culture conditions. This is in agreement with a previous report showing that GAPDH and 18s RNA mRNAs are not reliable references for qPCR assays in pharmacogenomics and toxicogenomics studies^83^. In contrast, RPL32 mRNA was shown to be a good reference for qPCR assays in mouse, rat and human post-infarction heart failure^80^ and mouse adipocytes and macrophages^57,82^. These results suggest that internal reference mRNA is probably different among the various tissues/cells tested.

Our study showed that most of the gene expression in human colon cancer cells was suppressed by high concentrations of gossypol (Table 3). Some of the *p* values were less than 5% threshold, suggesting their expression levels were statistically significant. By this standard, gossypol significantly decreased the expression of genes coding for mRNAs of CLAUDIN1, ELK1, FAS, GAPDH, IL2, IL8 and ZFAND5, but increased the expression of the gene coding for GLUT3 mRNA. These genes code for proteins involved in various biological processes and cancer development. CLAUDIN1 is a tight junction protein which is highly upregulated in colon cancer^49^. ELK1 is a transcription factor playing an important role in immunological response, which belongs to ETS protein family with the evolutionary conserved ETS domain stabilized by three key tryptophan residues interacting with DNA^42^. FAS is involved in the apoptotic system which is upregulated in human lung cancer cells (A549) by gossypol treatment for 12 h at 0.5 µmol/L (~ 0.26 µg/mL)^34^. GAPDH catalyzes the sixth step of glycolysis and serves to break down glucose for energy and carbon demands^83^. IL2 is an autocrine and paracrine growth factor involved in clonal T cell expansion, influences the magnitude and duration of an immune response, and contributes to the regulation of programmed cell death in T cells which is down-regulated by TTP through ARE-mediated mRNA decay^61^. IL8 induces chemotaxis in target cells, causes them to migrate toward the site of infection, and stimulates phagocytosis once they have arrived^63^. ZFAND5 enhances ARE-containing mRNA stability by competing with TTP for mRNA binding^84^. GLUT3 expressed specifically in neurons facilitates the transport of glucose across the plasma membranes of mammalian cells^85^.

Among the tested 55 genes, gossypol treatment inhibited the expression of well-known reference genes coding for GAPDH and RPL32 mRNAs (Fig. 3A). Among the genes reported to be regulated by gossypol in cancer cells^20,33–39^ and macrophages^40^, gossypol decreased the mRNA levels of BNIP3, CYP19A1, FAS, HUA, P53, PPARR and TNFSF10 genes in the human colon cancer cells (Fig. 3B). Gossypol decreased the mRNA levels of almost all of the DAGT, GLUT, TTP and IL gene families except GLUT3 in the cancer cells (Fig. 3C, D, 4A, B). The effect of gossypol on COX2, TNF and other proinflammatory cytokine mRNAs was not apparent (Fig. 4C), although it decreased the levels of a number of other TTP-regulated mRNAs coding for various functional proteins (Fig. 4D).

In our previous studies, gossypol significantly increased the expression of DGAT and HuR mRNAs in mouse RAW264.7 macrophages^40,56^. However, DGAT and HuR mRNAs were decreased by gossypol in the human colon cancer cells. Similarly, gossypol decreased FAS mRNA in colon cancer cells reported here but increased its expression in human lung cancer cells reported previously^34^. Finally, the mRNA levels of TTP family genes were shown here to be decreased in the colon cancer cells but reported to be increased in mouse macrophages^86^. These discrepancies might reflect the different responses of normal mouse macrophages and different human cancer cells to gossypol treatment.

This study provides evidence for the toxic effects of gossypol on cell viability and its effects on down-regulation of many gene expression at the mRNA level in the human colon cancer cells. However, there are a few limitations involved in the study which should be addressed in future study. First, the findings were derived from one colon cancer cell line (COLO225). It could be valuable to expand the scope of research with multiple cancer cell lines. Second, the general pattern of gossypol on decreasing mRNA levels of numerous genes was evident. However, the dosage effect of gossypol on mRNA levels was not strong and the standard deviations were large in many cases, probably due to extremely sensitive qPCR assays and many factors affecting the results from extracellular gossypol application, cell harvest, RNA extraction, cDNA synthesis to qPCR analysis. Third, it was a firm conclusion that gossypol negatively regulated many gene expression at the mRNA levels in the colon cancer cells. However, it could be great addition to confirm mRNA data at the protein level. Even though positive correlations between mRNA levels and protein levels are not guaranteed as shown in many publications, it is still valuable to perform experiments at the protein levels. Finally, this study provides evidence for gossypol’s toxic effects on cell viability and gene expression at the mRNA level in the human colon cancer cells. However, there is no functional analysis of gossypol affecting any intermediate steps between mRNA changes and cell viability. All these aspects are all worth of further investigation.

In conclusion, this study showed that gossypol significantly reduced the viability of human colon cancer cells. We further showed that BCL2 mRNA was the most stable among the 55 mRNAs in human colon cancer cells. Gossypol decreased the mRNA levels of DGAT, GLUT, TTP, ILs families and a number of previously reported genes. In particular, gossypol significantly suppressed the expression of the genes coding for CLAUDIN1, ELK1, FAS, GAPDH, IL2, IL8 and ZFAND5 mRNAs, but enhanced the expression of the gene coding for GLUT3 mRNA. This study provided evidence for potentially increasing the value of cottonseed by using cottonseed-derived gossypol as a health intervention agent.

## Materials and methods

### Colon cancer cell line

Human colon cancer cell line (COLO 205-ATCC CCL-222) was purchased from American Type Culture Collection (Manassas, VA) and kept under liquid nitrogen vapor in a Cryogenic Storage Vessel (Thermo Fisher Scientific, Waltham, MA). The cells were maintained at 37 °C in a humidified incubator with 5% CO_2_ in RPMI-1640 medium (Gibco, Life Technologies) supplemented with 10% (v:v) fetal bovine serum, 0.1 million units/L penicillin, 100 mg/L streptomycin, and 2 mmol/L L-glutamine.

### Chemicals, reagents and equipment

The chemicals, reagents and equipment were described essentially as previously^56^. Gossypol (molar mass: 518.56 g/mol) was purified from cottonseed by HPLC and purchased from Sigma (St. Louis, MO). Gossypol stock was prepared in 100% DMSO at 10 mg/mL (approximately 19.2 mM). Cell cytotoxicity reagent (MTT based-In Vitro Toxicology Assay Kit) and DMSO were from Sigma. Tissue culture reagents (RPMI-1640, fetal bovine serum, penicillin, streptomycin, L-glutamine) were from Gibco BRL (Thermo Fisher). Tissue culture incubator was water jacket CO_2_ incubator, Forma Series II, Model 3100 Series (Thermo Fisher). Tissue culture workstation was Logic + A2 hood (Labconco, Kansas City, MO). Tissue culture plastic ware (flasks, plates, cell scraper) was from CytoOne (USA Scientific, Ocala, FL). Cell counting reagent (trypsin blue dye), slides (dual chamber), counter (TC20 Automatic Cell Counter) and microscope (Zoe Florescent Cell Imager) were from Bio-Rad (Hercules, CA). Microplate spectrophotometer (Epoch) was from BioTek Instruments (Winooski, VT).

### Cell culture and chemical treatment

The basic cell culture protocol was following previous procedures^51,68,87^. Cancer cells were dissociated from the T-75 flask with 0.25% (w/v) trypsin-0.53 mM EDTA solution, stained with equal volume of 0.4% trypsin blue dye before counting the number of live cells with a TC20 Automatic Cell Counter. Cancer cells (0.5 mL) from trypsin-dissociated flasks were subcultured at approximately 1 × 10^5^ cells/mL density in 24-well tissue culture plates. The cancer cells were routinely observed under a Zoe Florescent Cell Imager before and during treatment. Cancer cells were treated with 0, 0.1, 0.5, 1, 5, 10, 50 and 100 µg/mL (corresponding to 0, 0.19, 0.96, 1.92, 9.6, 19.2, 96 and 192 µM) of gossypol for 2, 4, 8 and 24 h (“0” treatment corresponding to 1% DMSO in the culture medium, the vehicle control for the experiment). These gossypol concentrations were in the range of previously published concentrations for gossypol (up to 100 µM)^18,19,88^, (-) gossypol (up to 100 µM)^30^, apogossypolone (up to 40 µM)^89^, and gossypol derivatives (IC_50_ concentrations of 6–28 µM)^31^.

### Cell viability assay

Cell cytotoxicity was determined with the MTT based-In Vitro Toxicology Assay Kit^76^. Cancer cells in 96-well plates (12 wells/treatment) were treated with gossypol and incubated at 37 °C, 5% CO_2_ for 2, 4, 8 and 24 h. The cell media were added with 50 µL of MTT assay reagent (thiazolyl blue tetrazolium bromide) and incubated at 37 °C, 5% CO_2_ for 2 h before adding 500 µL MTT solubilization solution to each well, shaken at room temperature overnight. The color density in the wells was recorded by Epoch microplate spectrophotometer at A570.

### qPCR primers and probes

A total of 55 genes were selected for qPCR analysis. The selection of genes was based on the literature showing those gene expression regulated by gossypol in cancer cells^20,33–39^ and macrophages^40^ or regulated by ZFP36/TTP in tumor cells^41–49^ and macrophages^50,51^ (Table 1). RNA sequences were obtained from the National Center for Biotechnology Information (NCBI)’s non-redundant protein sequence databases (http://blast.ncbi.nlm.nih.gov/Blast.cgi). The qPCR primers were designed using Primer Express software (Applied Biosystems, Foster City, CA) and synthesized by Biosearch Technologies, Inc. (Navato, CA). The names of mRNAs and their nucleotide sequences (5′ to 3′) of the forward primers and reverse primers, and corresponding references are described in Table 1.

### RNA isolation and cDNA synthesis

The methods for RNA isolation and cDNA synthesis were essentially as described^56^. Human colon cancer cells in 24-well plates (triplicate) treated with various concentrations of gossypol for 8 h, a treatment showing significant reduction on cell viability (Fig. 2). The dishes were washed twice with 1 mL 0.9% NaCl and lysed directly with 1 mL of TRI_ZOL_ reagent (Invitrogen, Carlsbad, CA, USA). RNA was isolated according to the manufacturer’s instructions without DNase treatment and stored in -80 °C freezer. RNA concentrations were quantified with an Implen NanoPhotometer (Munchen, Germany). The cDNAs were synthesized from total RNA using SuperScript II reverse transcriptase. The cDNA synthesis mixture (20 μL) contained 5 μg total RNA, 2.4 μg oligo(dT)_12–18_ primer, 0.1 μg random primers, 500 μM dNTPs, 10 mM DTT, 40 u RNaseOUT and 200 u SuperScript II reverse transcriptase in 1X first-strand synthesis buffer (Life Technologies, Carlsbad, CA). The cDNA synthesis reaction was performed at 42 °C for 50 min. The cDNA was stored in − 80 °C freezer and diluted with water to 1 ng/µL before qPCR analyses.

### Quantitative real-time PCR analysis

The qPCR assays followed the MIQE guidelines: minimum information for publication of quantitative real-time PCR experiments^90^. The qPCR assays were described in details previously^54,82,91,92^. SYBR Green qPCR reaction mixture (12.5 μL) contained 5 ng of total RNA-derived cDNA, 200 nM each of the forward primer and reverse primer, and 1 × iQ SYBR Green Supermix (Bio-Rad Laboratories, Hercules, CA). The reactions in 96-well clear plates sealed by adhesives were performed with CFX96 real-time system-C1000 Thermal Cycler (Bio-Rad Laboratories). The thermal cycle conditions were as follows: 3 min at 95 °C, followed by 40 cycles at 95 °C for 10 s, 65 °C for 30 s and 72 °C for 30 s. BCL2 mRNA was selected as the internal reference based on its minimal variation of gene expression among the 55 genes tested in the colon cancer cells (see “Results” for details). Ribosome protein 32 (RPL32) and glyceraldehyde-3-phosphate dehydrogenase (GAPDH) mRNAs were widely used as the reference mRNAs in the qPCR analyses^87^ but they were not suitable for qPCR analysis for this cell type (see “Results” for details). TaqMan qPCR assay was used to confirm some SBYR Green qPCR assays using identical conditions as described previously^82^.

### Data analysis and statistics

The 2^−Δ*CT*^ and 2^−ΔΔ*CT*^ method of relative quantification was used to determine the fold change in expression^93^. This was done by first normalizing the resulting threshold cycle (*C*_*T*_) values of the target mRNAs to the *C*_*T*_ values of the internal control BCL2 mRNA in the same cells (Δ*C*_*T*_ = *C*_*T*Target_ − *C*_*T*Bcl2_). Gossypol-treated qPCR Δ*C*_*T*_ values was further normalized with DMSO qPCR Δ*C*_*T*_ values in the same cells (ΔΔ*C*_*T*_ = Δ*C*_*T*Gossypol_ − Δ*C*_*T*DMSO_). The fold change in expression was then obtained (2^−ΔΔ*CT*^). The data in the figures and tables represent the mean and standard deviation of various independent samples: Figure 2 (*n* = 12), Figs. 3 and 4 (*n* = 3), Tables 2 and 4 (*n* = 24), Table 3 (*n* = 3). They were analyzed by statistical analysis using ANOVA with SigmaStat 3.1 software (Systat Software). Multiple comparisons among the treatments with different concentrations of gossypol were performed with Student–Newman–Keuls method and Tukey test^87^.

## Abbreviations

DMSO: Dimethylsulfoxide
LPS: Lipopolysaccharides
MTT: 3-[4,5-Dimethylthiazol-2-yl]-2,5-diphenyl tetrazolium bromide
TTP: Tristetraprolin
ZFP36: Zinc finger protein 36
